# Neurotransmitter signaling in molecular and behavioral immune responses to pathogens in *C. elegans*

**DOI:** 10.1128/mmbr.00064-25

**Published:** 2025-09-11

**Authors:** Benson Otarigho, Alejandro Aballay

**Affiliations:** 1 Department of Genetics, The University of Texas MD Anderson Cancer Center 271626, Houston, Texas, USA; 2 Department of Microbiology and Molecular Genetics, McGovern Medical School at UTHealth, Houston, Texas, USA; University of Wisconsin-Madison, Madison, Wisconsin, USA

**Keywords:** immunity, avoidance behavior, serotonergic, dopaminergic, GABAergic, glutamatergic signaling, neurotransmitters

## Abstract

Neurotransmitter signaling pathways play major roles in both molecular and behavioral defenses against pathogen invasion, shaping the ability of *Caenorhabditis elegans* to sense and respond to environmental challenges. Given the conservation of neurotransmitter signaling pathways, their understanding may not only provide insights into the neurobiology of *C. elegans* but also has broader implications for our understanding of neural-immune interactions and host defense mechanisms in higher organisms. In this review, we discussed the literature on various neurotransmitter signaling pathways, including serotonergic, dopaminergic/octopaminergic, GABAergic, and glutamatergic pathways, and how these pathways modulate molecular and behavioral immune defense against pathogens.

## INTRODUCTION

Host survival against invading pathogens relies on a suite of well-developed strategies that encompass both molecular and behavioral defenses. In *Caenorhabditis elegans*, a rich repertoire of neurotransmitters modulates these responses, acting through defined mechanisms to influence immune activation and behavioral adaptation. *C. elegans* has emerged as a powerful model for studying neural-immune communication and host-pathogen interactions, owing to its simple and well-characterized nervous and immune systems, as well as its natural susceptibility to a broad range of microbial pathogens (1). These include *Pseudomonas aeruginosa*, *Staphylococcus aureus*, *Enterococcus faecalis*, *Candida albicans*, *Serratia marcescens*, enteropathogenic *Escherichia coli* (EPEC), and *Microbacterium nematophilum* (1–3). These pathogens use diverse virulence strategies such as exotoxin secretion, pore-forming toxins, oxidative stress induction, and proteolytic enzymes, leading to outcomes ranging from acute lethality to chronic immune activation (2–4). This diversity enables the dissection of conserved molecular immune pathways and adaptive behavioral responses.

The genetic tractability and optical transparency of *C. elegans* facilitate high-resolution, *in vivo* analysis of cellular, tissue-specific, and organismal responses (1, 3, 5). Importantly, many neurotransmitter signaling systems, including those involving dopamine, octopamine, serotonin, acetylcholine, and gamma-aminobutyric acid (GABA), are conserved between *C. elegans* and higher organisms (6), providing a platform for potential cross-species insights. Studies in *C. elegans* have revealed how these neurotransmitters modulate immune defenses through canonical signaling pathways and behavioral outputs (7–13). This review examines how these pathways shape both molecular and behavioral immune defenses in *C. elegans* and discusses potential broader implications of these findings for understanding neurotransmitter-regulated immunity across animals.

## OVERVIEW OF MAJOR NEUROTRANSMITTERS’ BIOSYNTHESIS AND FUNCTIONS

**Fig 1:**
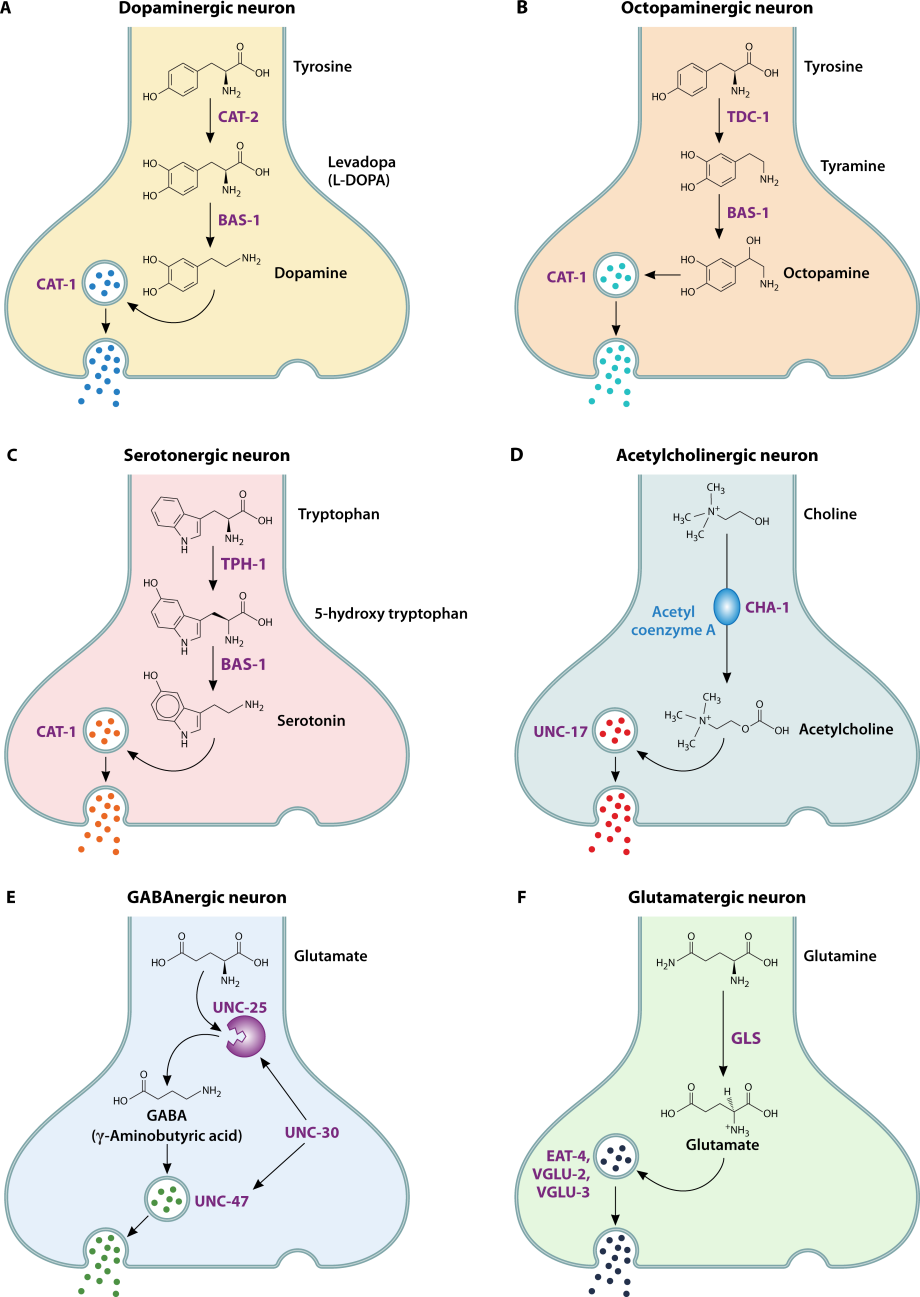
Biosynthesis of neurotransmitters known to participate in the immune response in *C. elegans*. (A) *De novo* synthesis of dopamine from the amino acid tyrosine and conversion to L-DOPA by tyrosine hydroxylase, which is encoded by the *cat-2* gene in *C. elegans*. L-DOPA is converted to dopamine and requires a biogenic amine synthase enzyme encoded by the *bas-1* gene in *C. elegans*. Dopamine is packaged and transported in presynaptic vesicles by the vesicular monoamine transporter, which is encoded by the *cat-1* gene in *C. elegans*. (B) *De novo* synthesis of octopamine from tyrosine and conversion to tyramine by tyrosine decarboxylase in *C. elegans* is catalyzed by the *tdc-1* gene. Tyramine is then converted to octopamine by tyramine beta-hydroxylase in *C. elegans*, which is encoded by the *tbh-1* gene. Octopamine is packaged and transported in presynaptic vesicles by the vesicular monoamine transporter, which is encoded by the *cat-1* gene in *C. elegans*. (C) *De novo* synthesis of serotonin from tryptophan and its conversion to 5-hydroxy tryptophan by tryptophan hydroxylase (tph)-type enzymes in *C. elegans*, which are encoded by the *tph-1* gene. TPH is converted to serotonin and requires a biogenic amine synthase enzyme encoded by the *bas-1* gene in *C. elegans*. Serotonin is packaged and transported in presynaptic vesicles by the vesicular monoamine transporter, which is encoded by the *cat-1* gene in *C. elegans*. (D) *De novo* synthesis of acetylcholine from choline by choline acetyltransferase (ChAT) in *C. elegans*; ChAT is encoded by the *cha-1* gene. In *C. elegans*, acetylcholine is packaged and transported in presynaptic vesicles, which are encoded by the *unc-17* gene. (E) *De novo* synthesis of GABA from glutamate by GAD results in the packaging of GABA into vesicles by VGAT in *C. elegans*, and GABA is encoded by the unc-25 gene. GABA is packaged and transported in presynaptic vesicles, which are encoded by the *unc-47* gene. Both *unc-25* and *unc-47* are transcriptionally regulated by unc-30 in *C. elegans*. (F) The *de novo* synthesis of glutamate from glutamine by the glutaminase (GLS) enzyme in *C. elegans* is catalyzed by the *glna-1*, *glna-2*, and *glna-3* genes. Glutamate is packaged and transported in presynaptic vesicles, which are encoded by the *eat-4*, *vglu-2*, and *vglu-3* genes in *C. elegans*.

In *C. elegans*, dopamine and octopamine are synthesized from tyrosine via distinct enzymatic pathways. Dopamine is produced by tyrosine hydroxylase (*cat-2*) and biogenic amine synthase (*bas-1*), while octopamine is generated through tyrosine decarboxylase (*tdc-1*) and tyramine beta-hydroxylase (*tbh-1*) (14). Functionally, these neurotransmitters influence behaviors such as locomotion, cognition, stress response (15–19), and immunity (10, 20–25). Serotonin is synthesized from tryptophan by tryptophan hydroxylase (*tph-1*) (19, 26, 27) and plays key roles in feeding, locomotion, and immune modulation. Its reuptake and degradation are regulated by serotonin transporters and monoamine oxidase (19, 28, 29). Acetylcholine, produced by choline acetyltransferase (*cha-1*) (27, 30), is involved in motor control (30, 31), immune responses, and other physiological processes (32–34). GABA is synthesized from glutamate by glutamic acid decarboxylase (GAD), encoded by *unc-25* (35–37), and controls locomotion and digestion (36–38). Glutamate, synthesized from glutamine (27, 39), is crucial for chemosensory, mechanosensory, and stress responses (40). The biosynthetic pathways of these neurotransmitters are shown in Fig. 1.

Growing evidence indicates that neurotransmitter signaling plays a critical role in regulating both molecular and behavioral immunity (11–13, 41). To discuss this dual role in *C. elegans*, we highlight key studies demonstrating how these signaling pathways influence immune gene expression and shape host defense responses during pathogen exposure.

## ROLE OF DOPAMINERGIC SIGNALING IN MOLECULAR IMMUNE DEFENSE

Dopaminergic signaling has been associated with immune function in several organisms (42, 43), including *C. elegans* (10, 21, 22). In mammals, the role of dopamine in immune regulation was discovered only recently (44). Earlier research on dopaminergic signaling in *C. elegans* and other organisms primarily focused on behavioral roles (45), until studies revealed that dopamine also regulates molecular immunity in *C. elegans* via the D1-like dopamine receptor DOP-4 (22). In this context, dopamine acts through CEP neurons to inhibit immunity, requiring DOP-4 activity in downstream ASG sensory neurons (Table 1). This signaling suppresses *C. elegans* survival against pathogens by inhibiting the activation of the PMK-1/p38 MAPK immune pathway (22).

**TABLE 1:** Neurons involved in neurotransmitter-mediated immune responses in *C. elegans*

Neuron	Location/type	Primary function in context	Associated neurotransmitter(s)	References
CEP	Head/sensory neuron	Dopamine release; modulates learned bacterial avoidance and immunity via DOP-4	Dopamine	(22)
ADE	Head/sensory neuron	Involved in learned pathogen avoidance	Dopamine	(46)
ASG	Head/sensory neuron	Receives dopaminergic signal to suppress p38/PMK-1 immune pathway; also GABA-regulated	Dopamine, GABA	(22)
ADF	Head/chemosensory neuron	Regulates serotonin synthesis and dopamine-mediated avoidance; modulates insulin-like pathway	Dopamine, Serotonin	(47)
RIC	Head/interneuron	Octopaminergic; transiently activated by non-pathogens; downregulated during pathogen exposure	Octopamine	(20)
ASH	Head/sensory neuron	Mediates suppression of immune gene expression via OCTR-1	Octopamine	(21, 48)
ASI	Head/sensory neuron	Works with ASH in OCTR-1 signaling to suppress UPR and immunity	Octopamine	(21)
AWB	Head/chemosensory neuron	Regulates serotonin synthesis; promotes aversive learning and insulin signaling for behavioral immunity	Serotonin	(47)
RIM	Head/interneuron	Non-cholinergic neuron receiving input from ACC-4; regulates immune gene expression via Wnt signaling	Acetylcholine	(22)
AWA	Head/chemosensory neuron	Mediates experience-dependent aversive behavior via cholinergic and GPCR signaling	Acetylcholine	(49)
AWC	Head/chemosensory neuron	Works with AWA in aversive learning; expresses nAChRs and Gα proteins	Acetylcholine	(49)

Additional studies have shown that ADF neurons, which regulate dopamine synthesis, inhibit the expression of innate immune genes and enhance behavioral immune responses via the DAF-16/insulin-like growth factor 1 (IGF-1) signaling pathway (47). Dopamine has also been shown to positively regulate molecular immune defense against *Salmonella enterica* infection via the PMK-1/p38-MAPK and DAF-16/IGF-1 pathways (23). Furthermore, the dopamine transporter DAT-1 plays a role in regulating the molecular immune response to the fungal pathogen *C. albicans* (50).

**Fig 2:**
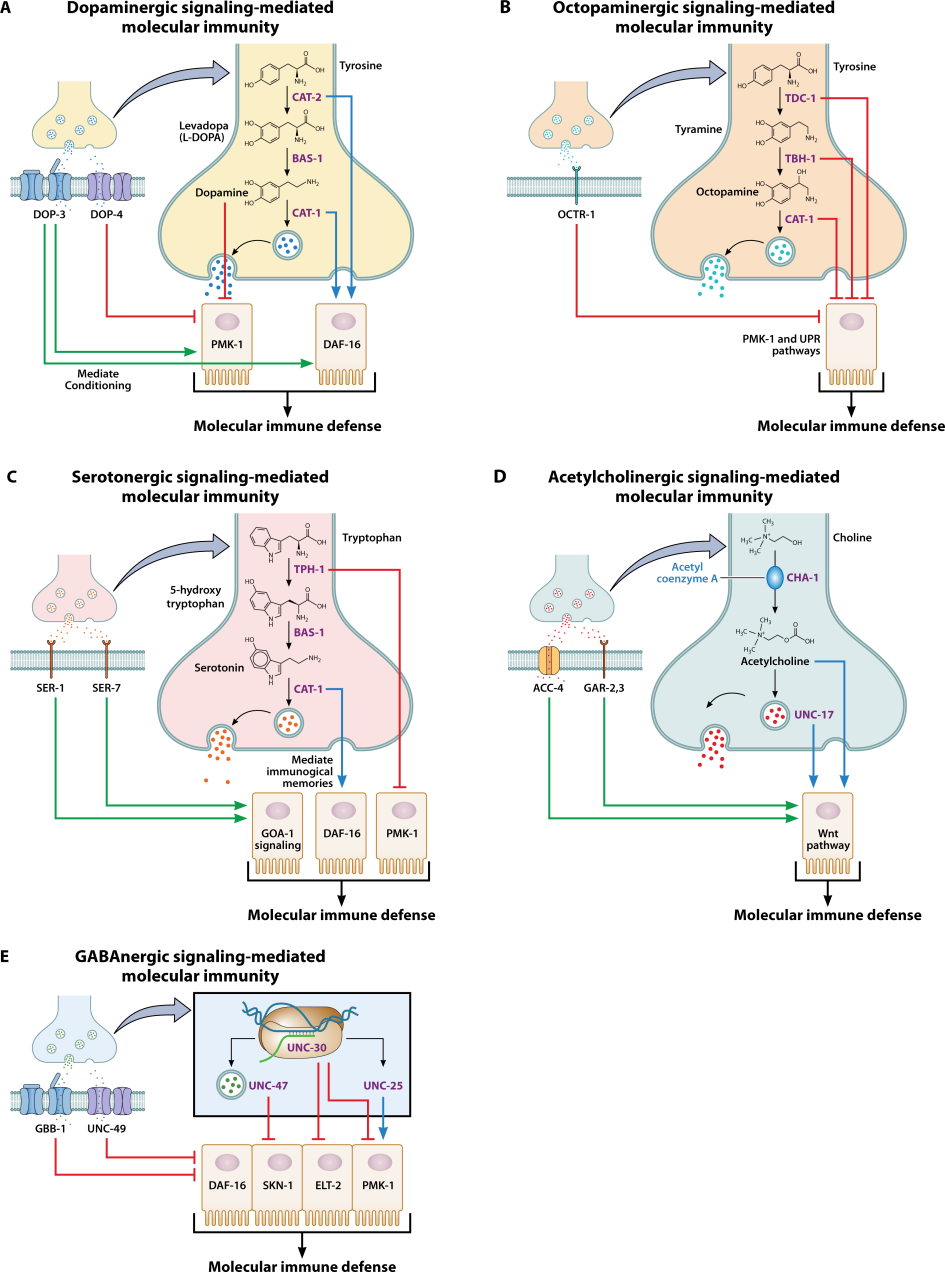
Neurotransmitter signaling modulates molecular immune defense in *C. elegans*. (A) Dopaminergic signaling-mediated immune defense via DOP-4, DOP-3, CAT-1, and CAT-2. (B) Octopaminergic signaling-mediated immune defense via OCTR-1, TDC-1, TBH-1, and CAT-1. (C) Serotonergic signaling-mediated immune defense via TPH-1, CAT-1, SER-1, and SER-7. (D) Acetylcholinergic signaling-mediated immune defense via UNC-17, ACC-4, GAR-2, and GAR-3. (E) GABAergic signaling-mediated immune defense via UNC-30, UNC-25, UNC-47, UNC-49, and GBB-1.

In another study, pre-exposure of wild-type *C. elegans* to the lethal bacterial pathogen EPEC resulted in increased survival during subsequent infection. This protective effect was mediated by the PMK-1/p38 MAPK and DAF-16/IGF-1 pathways (51). Notably, mutation of the *dop-3* gene, which encodes a dopamine receptor, impaired this conditioning response (51), highlighting the contribution of dopaminergic signaling to molecular immune adaptation. Mutations in *cat-1* or *cat-4*, genes required for dopamine synthesis and transport, were also shown to disrupt immunological memory (long-term immune response from prior exposure, independent of adaptive immunity) upon pathogen pre-exposed (47). Together, these findings establish dopamine as a key neuromodulator of molecular immunity in *C. elegans*, regulating both neuronal and epithelial responses via defined receptor-mediated pathways (Fig. 2A). These studies underscore dopamine’s broader role in linking neural cues to immune outcomes during pathogen challenge.

### Role of dopaminergic signaling in behavioral immune defense

Mutants defective in dopamine signaling have been shown to exhibit altered pathogen-mediated behavioral immune responses in *C. elegans*. For instance, mutations in *cat-2* and *dat-1* impair learned bacterial avoidance, highlighting the role of dopaminergic modulation in behavioral immunity (46). Cell-specific rescue experiments suggest that dopamine released from CEP and ADE neurons governs this learned avoidance (Table 1), a process further disrupted by mutations in dopamine receptor genes such as *dop-1, dop-2*, and *dop-3* (46).

**Fig 3:**
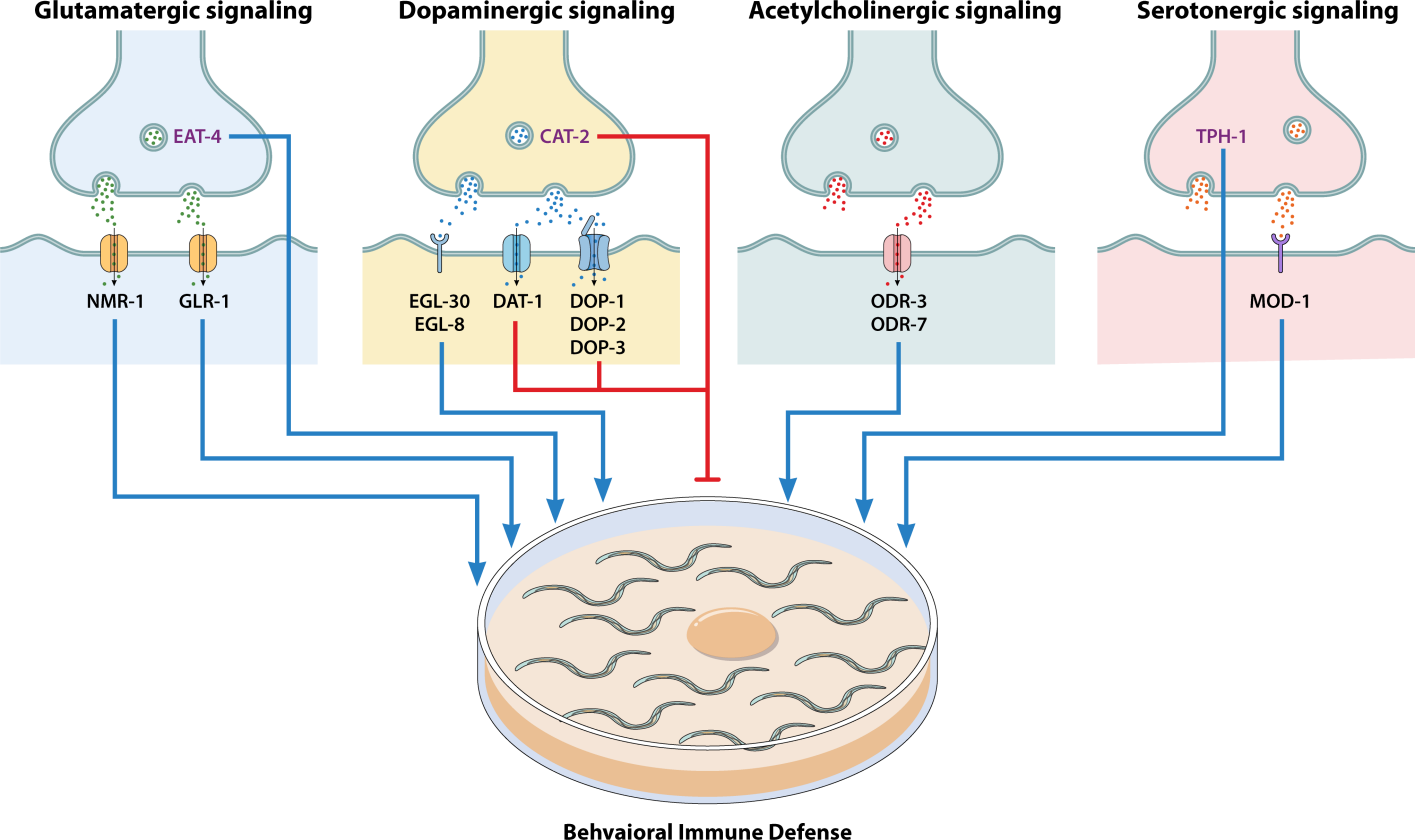
Neurotransmitter signaling-mediated behavioral immune defense against pathogens. The black activation arrows indicate enhanced behavioral immunity, while the green inhibition arrows indicate decreased behavioral immunity via immunological memories.

Yan et al. (47) demonstrated that dopaminergic signaling via ADF neurons promotes pathogen avoidance via the downstream DAF-16/IGF-1 pathway. In addition to dopaminergic signaling, the G protein EGL-30 (Gαq) has been shown to regulate the behavioral immune response against *P. aeruginosa*, acting as a G protein subunit that mediates neurotransmitter signals influencing host defense (49). These findings indicate that dopamine influences not only molecular immunity but also pathogen-driven behavioral adaptations such as learned bacterial avoidance (Fig. 3). Collectively, this supports a dual role for dopaminergic signaling in coordinating both sensory neurons’ activities and behavioral immune strategies.

## ROLE OF OCTOPAMINERGIC SIGNALING IN MOLECULAR IMMUNE DEFENSE

Like dopaminergic signaling, octopaminergic signaling modulates the molecular immune response to pathogen infection (20, 21, 52). The neuronal octopamine receptor OCTR-1 suppresses immune activation acting in ASH and ASI sensory neurons to downregulate the expression of PMK-1-controlled genes and unfolded protein response genes in nonneuronal tissues (21). Another study demonstrated that octopamine acts as an endogenous ligand for OCTR-1 in immune regulation via RIC neurons (20), which are inhibited in the presence of pathogens but transiently activated by nonpathogenic bacteria (20). In addition to its immune-suppressive role, OCTR-1 contributes to the restoration of protein homeostasis after infection (48). These findings establish octopamine as a key immunosuppressive signal (Fig. 2B), illustrating how specific neurotransmitters and neurons can limit immune activation and help maintain physiological balance during environmental stress.

## ROLE OF SEROTONERGIC SIGNALING PATHWAY IN MOLECULAR IMMUNE DEFENSE

The serotonergic signaling pathway has been shown to modulate molecular immune defense in a wide range of animal models (10, 23, 53–58). In *C. elegans*, several mechanisms underlying the role of serotonin signaling in the molecular immune response have been observed in the gut. An evaluation of the effects of serotonin on the innate immune response in *C. elegans* demonstrated that excess serotonin modulates the activation of the GTPase RHO-1 (59). Moreover, the effects of increased serotonin levels on the activation of this small GTPase imply that serotonin has the potential to modulate genetic circuits following different infections. This work further demonstrated that serotonin suppresses the molecular immune response and limits the rate of pathogen clearance (59).

The role of serotonin receptors in the *C. elegans* immune response was also investigated using mutants in the genes encoding the serotonin receptors SER-1 and SER-7 (50, 60, 61). SER-1 and SER-7 are G protein serotonin receptors homologous to human 5-hydroxytryptamine receptor 2A and 5-hydroxytryptamine receptor 7, respectively. In the rectal epithelium, serotonin signaling via the SER-1 and SER-7 receptors activates GOA-1, a Gαo subunit, leading to suppression of both the Dar (distended anal region) phenotype and immune response to pathogens (59). Further study demonstrated that AWB chemosensory neurons, which are known to modulate the synthesis of serotonin, decrease the expression of innate immunity-associated genes via the downstream insulin-like pathway (47). Consistently, global transcriptomic analyses of *C. elegans* animals exogenously treated with serotonin revealed significant dysregulation of defense and immune response genes (62). Similar to dopaminergic signaling, serotonergic signaling is also required for the modulation of immunological memory upon preexposure to pathogens (47). Taken together, these studies reveal that serotonin actively modulates intestinal immune signaling and gene expression in response to pathogens (Fig. 2C). Its regulatory function reflects a critical link between neuronal serotonin signaling and epithelial immune homeostasis.

### Role of serotonergic signaling in behavioral immune defense

Serotonin plays a key role in behavioral immunity by influencing aversive learning (learned bacterial avoidance) and memory processes that allow *C. elegans* to adaptively avoid pathogens. It modulates pathogen-induced behavioral responses, particularly through associative learning mechanisms (47, 63–65). Research has shown that the chemosensory neuron AWB regulates the synthesis of serotonin. This regulation subsequently reduced the expression of genes associated with innate immunity, resulting in an enhanced behavioral immune response mediated by the downstream insulin-like pathway (Table 1) (47). A separate investigation demonstrated that when *C. elegans* is exposed to pathogenic bacteria, serotonin levels increase in ADF chemosensory neurons through both transcriptional and posttranscriptional pathways (63). Findings from Zhang et al. also established that serotonin exerts its effects via MOD-1, a serotonin-gated chloride channel expressed in sensory interneurons, to facilitate aversive learning. Elevated serotonin levels may, therefore, serve as an adverse reinforcing stimulus during pathogenic infection (63). Furthermore, it has been demonstrated that the *tph-1* gene, which encodes tryptophan hydroxylase and plays a critical role in the rate-limiting step of serotonin biosynthesis, enhances the behavioral immune response to pathogens in *C. elegans* (65). In addition, serotonin plays a vital role in promoting aversive learning so that animals alter their olfactory preferences to avoid pathogenic bacteria in future encounters (63, 64). Recent findings further demonstrate that constitutive activation of the cytoprotective factor SKN-1/nuclear factor erythroid 2-related factor 2 (Nrf2) within the digestive and nervous system in *C. elegans* causes serotonin depletion, leading to a unique pathogen apathy where the worms fail to avoid the harmful bacterium *P. aeruginosa* (66). This study uncovers a novel neuroimmune pathway in which SKN-1-regulated serotonin signaling coordinates neuronal and intestinal responses to modulate behavioral immune response to pathogens, impacting host survival during infection (66). These studies demonstrate serotonin’s essential function in encoding pathogenic experiences and driving aversive behavioral strategies that promote survival (Fig. 3).

## ROLE OF ACETYLCHOLINE SIGNALING IN MOLECULAR IMMUNE DEFENSE

Acetylcholine signaling has emerged as a key regulator of immune gene expression in *C. elegans*, acting through both muscarinic and nicotinic acetylcholine receptors (nAChRs), particularly within the intestinal epithelium during bacterial infection. This role was first uncovered by Labed et al. (32), who demonstrated that cholinergic signaling modulates the innate immune response in the gut during *S. aureus* infection, revealing a previously unrecognized neuroimmune pathway. In this study, the researchers used RNA interference to screen 890 GPCR genes and found that silencing *gar-2* and *gar-3*, which encode muscarinic acetylcholine receptors, significantly reduced the expression of *S. aureus*-induced immune genes. Furthermore, treatment of wild-type animals with the ACh mimic arecoline or the muscarinic agonist oxotremorine increased the expression of immune genes. This effect was found to be reversible upon silencing *gar-2* or *gar-3*. Conversely, the muscarinic antagonist scopolamine inhibited the induction of immune genes by either *S. aureus* or arecoline (32).

Another study demonstrated the role of *C. elegans* ACC-4, a member of the acetylcholine receptor family, in immune activation triggered by abnormalities in the defecation motor program or pathogen infection (33). This work further showed that ACC-4 functions postsynaptically in noncholinergic RIM neurons to regulate the expression of several immune genes and facilitate a Wnt-mediated host immune response. Together, these findings highlight the critical role of acetylcholine signaling in regulating molecular immune responses in *C. elegans*, primarily through receptor-mediated pathways that activate intestinal defense mechanisms (Fig. 2D).

### Role of acetylcholine signaling in behavioral immune defense

Researchers have shown that the G protein α subunit EGL-30 and its downstream effectors EGL-8 and UNC-13 regulate the behavioral immune response to *P. aeruginosa* through acetylcholine signaling and activation of nAChRs. This EGL-30-mediated G protein signaling pathway functions in AWA and AWC neurons to mediate experience-dependent aversive behavior toward the pathogen (49). In this context, ODR-3 (Gαi) acts in AWC neurons, while ODR-7 functions in AWA neurons as a nuclear receptor required for maintaining AWA neuronal identity and regulating the expression of AWA-specific signaling molecules (49).

Similarly, McMullan et al. showed that exposure to pathogenic, but not avirulent, strains of *M. nematophilum* triggers an aversive behavioral response in *C. elegans* mediated by Gαq signaling in cholinergic motor neurons. Activation of this pathway enhances acetylcholine release and increases locomotion, allowing animals to leave the bacterial lawn and avoid infection. This demonstrates that acetylcholine signaling through Gαq not only regulates movement but also enables experience-dependent behavioral adaptation to pathogenic bacteria (67).

These studies suggest that acetylcholine signaling contributes to the organism’s ability to detect and behaviorally respond to pathogenic threats (Fig. 3). Its modulation of neuronal pathways and locomotion patterns further supports its dual role in both neuromuscular activity and behavioral immunity.

## ROLE OF GABAERGIC SIGNALING IN MOLECULAR IMMUNE DEFENSES

GABAergic neurons influence immune gene expression through neuroepithelial and neuromuscular interactions, revealing a neuroimmune axis in response to pathogens. GABAergic signaling has been shown to modulate the molecular immune response in *C. elegans* (11–13, 41). Experimental evidence indicates that the PITX transcription factor UNC-30, a homeodomain-containing transcription factor that is known to regulate the biosynthesis of GABA, regulates the molecular immune response to pathogens through the nervous system in *C. elegans*. UNC-30 was found to control immunity via the ELT-2/GATA transcription factor and PMK-1/p38 MAPK pathways, acting through ASG sensory neurons (11). Zheng et al. (13) further demonstrated that GABAergic neuromuscular junctions control immunity by inducing the insulin-like peptide INS-31 in the muscles, thereby suppressing the innate immune response in the intestine of *C. elegans*. More recently, GABAergic signaling between enteric neurons and intestinal smooth muscle was shown to promote gut defense through PMK-1. This effect occurs independently of DAF-16/IGF-1 and DBL-1/TGF-β and is mediated by the neuropeptide FLP-6, which is expressed and secreted by intestinal smooth muscle cells (41). The same research group also found that impaired GABAergic neurotransmission increases reactive oxygen species (ROS) and inhibits SKN-1/Nrf2 nuclear translocation, whereas exogenous GABA reduces ROS levels (12). Together, these studies demonstrate that GABAergic signaling serves as a crucial regulatory axis in neuroimmune communication, influencing both neuronal and intestinal immune responses in *C. elegans*. This highlights GABA’s role not only in neural inhibition but also in modulating innate immune pathways during infection. (Fig. 2E).

## ROLE OF GLUTAMATERGIC SIGNALING IN BEHAVIORAL IMMUNE DEFENSES

The involvement of glutamate signaling in regulating the pathogen-mediated behavioral immune response in *C. elegans* remained unknown until recently, when two separate studies elucidated its importance in this context (68, 69). Yu and Chang reported that glutamate transmission is pivotal for the behavioral adaptability to *P. aeruginosa*. Their study showed that deletion of the vesicular glutamate transporter gene *eat-4* impaired the mutant’s ability to exhibit either attraction or aversion toward *P. aeruginosa*. The study also showed that the AMPA-type glutamate receptor GLR-1 enhances the behavioral immune response against *P. aeruginosa*, with superoxide dismutase, SOD-1, acting downstream of GLR-1 in cholinergic motor neurons (68). In a separate study, the NMDA-type glutamate receptor NMR-1 was found to trigger pathogen-mediated behavioral immunity and to prime naïve *C. elegans* for aversive learning (69). Collectively, these findings establish glutamatergic signaling as a key regulator of behavioral immune responses in *C. elegans*, particularly in mediating aversive learning and experience-dependent avoidance of pathogens (Fig. 3). This underscores glutamate’s broader role in integrating environmental cues with adaptive behavioral strategies for host defense.

## CROSS-TALK AND CONVERGENCE OF NEUROTRANSMITTER SIGNALING PATHWAYS IN HOST DEFENSE

Although each neurotransmitter system influences immunity through distinct receptors and neuronal circuits, emerging evidence suggests substantial cross-talk and convergence among these pathways in *C. elegans* (23, 24, 47). For example, both dopamine and serotonin regulate behavioral immune responses such as learned pathogen avoidance, and both appear to act through common downstream effectors, including the insulin/IGF-1 signaling pathway and the PMK-1/p38 MAPK cascade (47). Similarly, GABA and acetylcholine modulate intestinal immune responses via transcriptional regulators like ELT-2/GATA and SKN-1/Nrf2, suggesting that neurotransmitter systems with distinct upstream origins can converge on common immune outcomes (11, 33, 41).

This growing recognition of pathway convergence highlights the importance of studying neurotransmitter signaling as part of an integrated neuroimmune network rather than in isolation. Future research should focus on identifying the molecular nodes where these pathways intersect, characterizing potential combinatorial effects, and understanding how cross-talk modulates host immunity in a context-dependent manner.

### Broader relevance of neurotransmitter signaling in molecular and behavioral immune responses

Beyond *C. elegans*, neurotransmitter pathways also regulate immunity and behaviors in other animal models, reinforcing conserved roles. In mammals, dopamine influences immune responses through D1- and D2-like receptors on immune cells and controls responses to aversive experiences, although its specific role in the context of pathogen aversion is not known (44, 70). Serotonin modulates immunity in both vertebrates and invertebrates, including oysters, and supports olfactory-guided pathogen avoidance and learning in insects such as *Drosophila* (57, 71, 72). Similarly, octopamine, which is functionally analogous to norepinephrine in *Drosophila*, regulates both immune activation and behavioral arousal during infection (73). In rodents, acetylcholine suppresses inflammation via α7 nAChRs and influences learning and stress-related behaviors (74). GABA signaling contributes to immune tolerance and regulates stress and anxiety-related behaviors (75). Glutamate, through its receptors, modulates cytokine release and plays a critical role in fear learning and memory formation in mammals (76). Together, these findings highlight the conserved role of neurotransmitter signaling in coordinating immune responses across species and underscore the value of *C. elegans* for comparative neural-immune signaling research.

## CONCLUSIONS

This review addresses the key roles of serotonergic, dopaminergic/octopaminergic, GABAergic, acetylcholinergic, and glutamatergic signaling in regulating both molecular and behavioral defenses in *C. elegans* against pathogen infections. The findings discussed here shed light on the role of neurotransmitter signaling in *C. elegans*, offering important insights into neural-immune cross-talks.

Given the conservation of the signaling pathways discussed here, the knowledge gained from *C. elegans* may help elucidate how neurotransmitters influence defense mechanisms in more complex organisms. Many of the neuromodulators described also regulate immune responses in mammals through homologous receptors and pathways. Since these signaling pathways are also implicated in numerous human conditions such as depression, schizophrenia, Parkinson’s disease, and other neuroimmune and inflammatory disorders (19, 77–79), studies in *C. elegans* may uncover mechanisms relevant to human health and disease. Further work may help define conserved neuroimmune principles that may inform new therapeutic strategies for infection, inflammation, and neuroimmune disorders.
